# Supplementary material for: Developing shared understanding of pre-eclampsia in Haiti and Zimbabwe using Theory of Change
Source: PLOS Glob Public Health. 2022 Dec 16;2(12):e0001352. doi: 10.1371/journal.pgph.0001352 (PMC10021157; doi:10.1371/journal.pgph.0001352)
Supplement: S2 Text — (DOCX) [file pgph.0001352.s002.docx]

**Supplementary materials**

Links to films on YouTube

Chwa Astryd (Astryd’s Choice) a collaboration between HAPPEE Partnership Project Haiti, Raw TV and Tom Besley

<https://www.youtube.com/watch?v=RL2Lsk-0nSY>

Rudo neVanhukadzi (For the love of women) a film by the HAPPEE Partnership Project Zimbabwe <https://youtu.be/kq00YPhhN-8>
