# Supplementary figures and images for: Developing shared understanding of pre-eclampsia in Haiti and Zimbabwe using Theory of Change
Source: PLOS Glob Public Health. 2022 Dec 16;2(12):e0001352. doi: 10.1371/journal.pgph.0001352 (PMC10021157; doi:10.1371/journal.pgph.0001352)

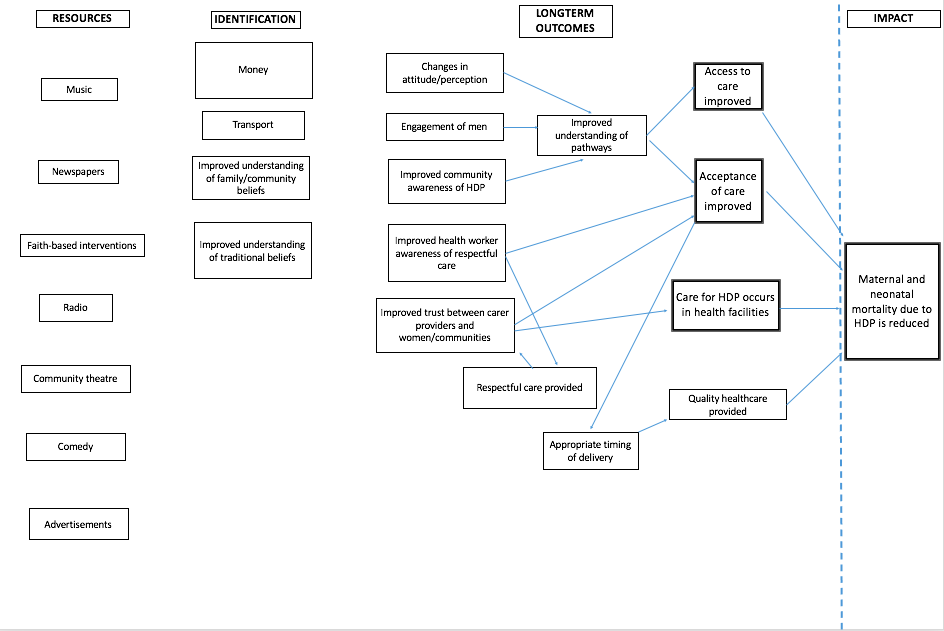

Supplement: S1 Fig — (TIFF) [file pgph.0001352.s003.tiff]

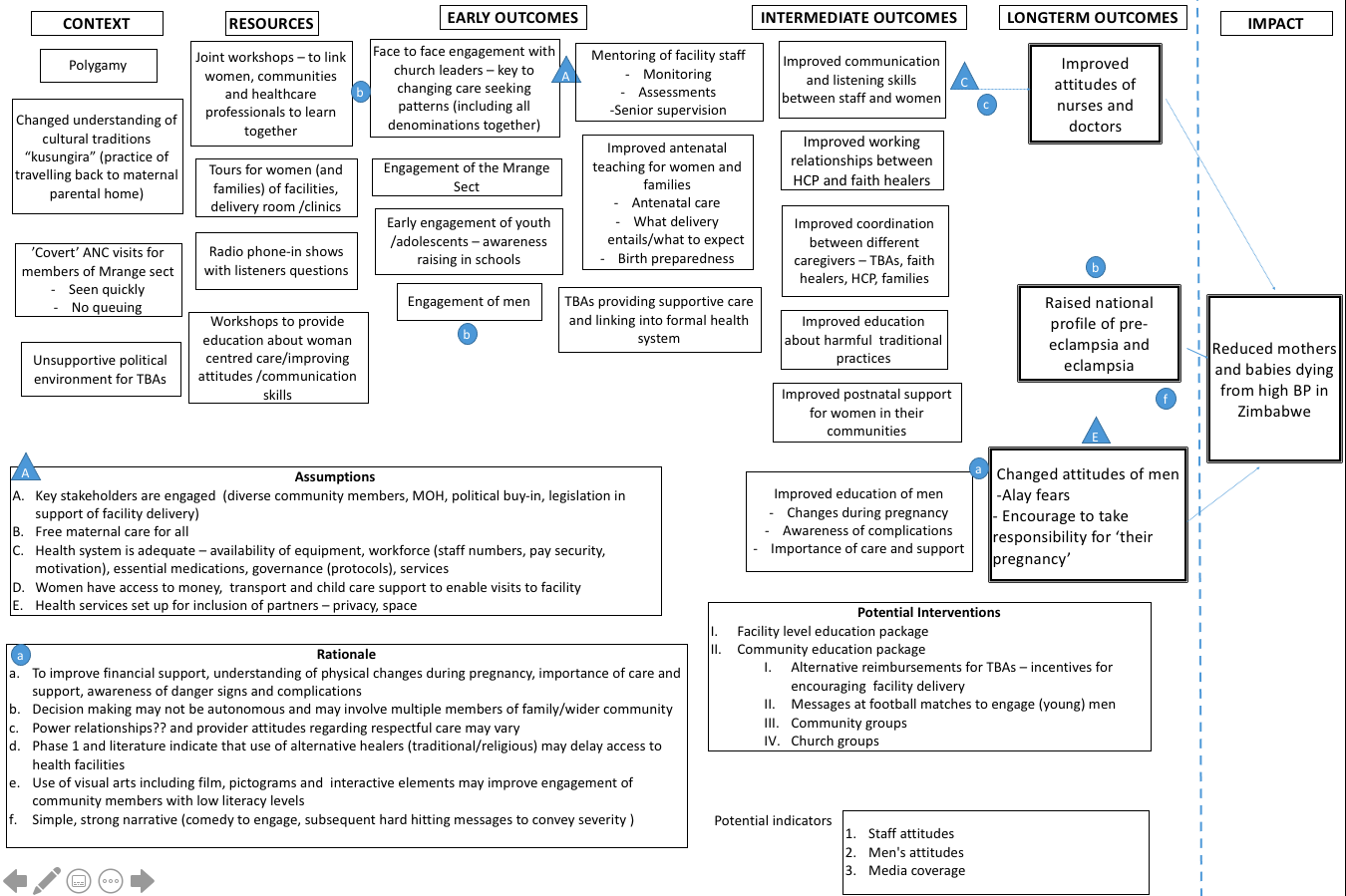

Supplement: S2 Fig — (TIFF) [file pgph.0001352.s004.tiff]

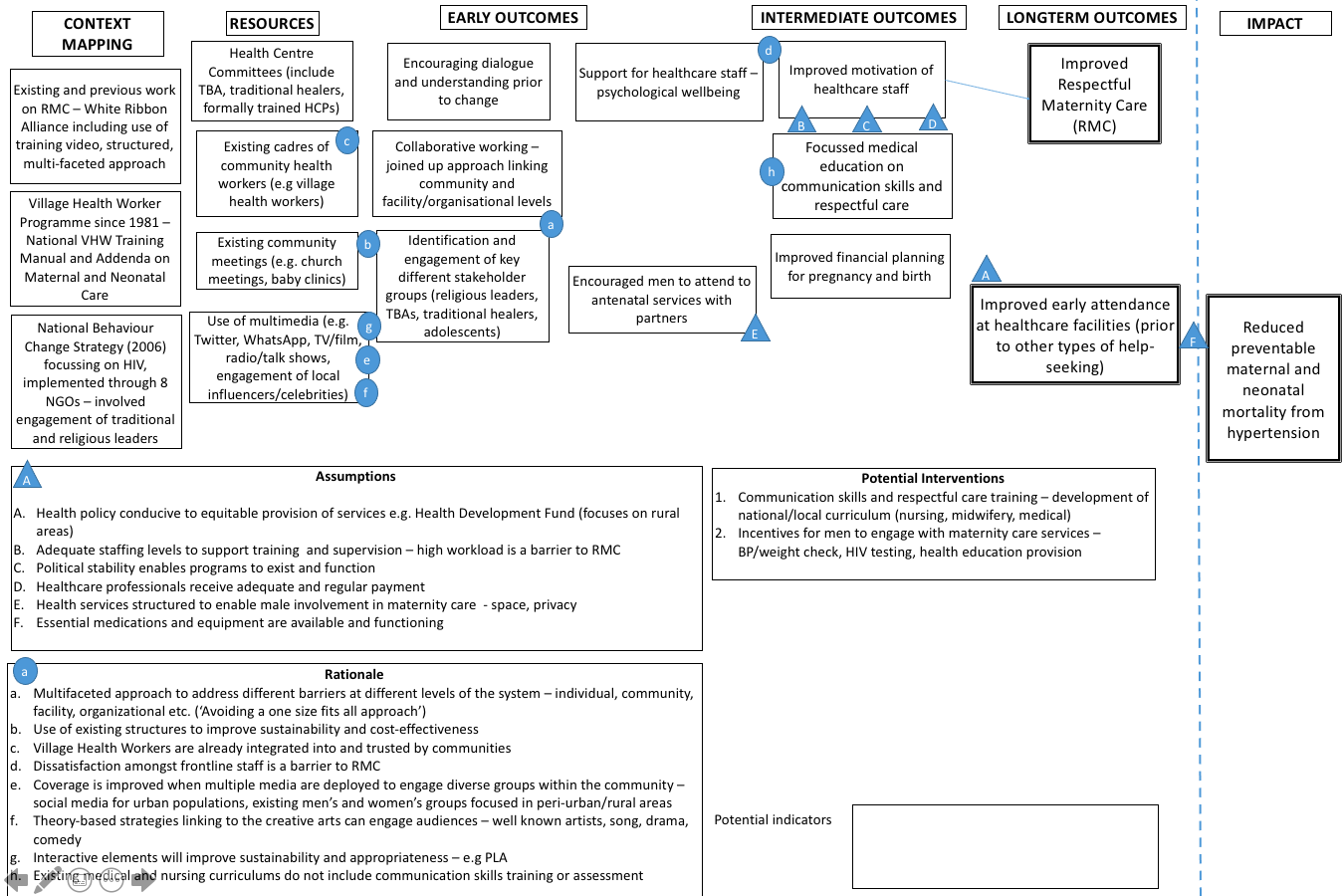

Supplement: S3 Fig — (TIFF) [file pgph.0001352.s005.tiff]
