# Supplementary material for: Developing shared understanding of pre-eclampsia in Haiti and Zimbabwe using Theory of Change
Source: PLOS Glob Public Health. 2022 Dec 16;2(12):e0001352. doi: 10.1371/journal.pgph.0001352 (PMC10021157; doi:10.1371/journal.pgph.0001352)
Supplement: Attachment — Submitted filename: Response to reviewers.docx [file pgph.0001352.s006.docx]

Dr Tanya Robbins

Department of Women and Children’s Health

School of Life Course Science

King’s College London

10^th^ Floor, North Wing

St Thomas’ Hospital

Westminster Bridge Road

London SE1 7EH

tanya.robbins@kcl.ac.uk

PLOS Global Public Health

August 12, 2022

Dear Editorial Team

I am pleased to resubmit our paper entitled ‘Developing shared understanding of pre-eclampsia in Haiti and Zimbabwe using Theory of Change’ for consideration for publication in *PLOS Global Public Health.* Thank you for the encouraging comments and suggestions for minor revisions. Please see below the point by point response to the reviewer’s and editorial comments.

Thank you for your consideration.

Sincerely,

Dr Tanya Robbins

Reviewer’s comments:

1. The reference to the COVID pandemic does not seem to fit well into the general flow of the manuscript. Please reframe this section.

This section has been reframed in response to the reviewer’s comment. See lines 102 - 108.

2. HAPPEE trial could be introduced earlier in the manuscript. When mentioned in line 149, there seems to be no background about what it is and why it is mentioned there.

The HAPPEE Partnership Project has been outlined in the introduction. See line 123.

3. The keywords contains ETHIOPIA which does not seem to be featuring in the manuscript at all.

This keyword was added in error and has now been removed.

4. The sentence with Ceiling of accountability as mentioned in line 133 is not easily understood. Please reframe.

This sentence has now been removed as does not directly relate to the Theory of Change developed as part of our project.

Journal requirements:

1. Please provided the full name of the IRB or ethics committee.

This work was a component of the HAPPEE (Humanities and Arts in Preventing Pre-eclampsia complications through community Engagement and Education) Partnership Project, approved by the Biomedical & Health Sciences, Dentistry, Medicine and Natural & Mathematical Sciences Research Ethics Subcommittee, King’s College London (LRS-17/18-5526).

See lines 174-177.

2. Please include a complete copy of PLOS’ questionnaire on inclusivity in global research in your revised manuscript. Our policy for research in this area aims to improve transparency in the reporting of research performed outside of researchers’ own country or community. The policy applies to researchers who have travelled to a different country to conduct research, research with Indigenous populations or their lands, and research on cultural artefacts. The questionnaire can also be requested at the journal’s discretion for any other submissions, even if these conditions are not met.  Please find more information on the policy and a link to download a blank copy of the questionnaire here: https://journals.plos.org/globalpublichealth/s/best-practices-in-research-reporting. Please upload a completed version of your questionnaire as Supporting Information when you resubmit your manuscript.

PLOS’ questionnaire on Inclusivity in global research has been added as Supporting Information in resubmission.

3. Please amend your detailed Financial Disclosure statement. This is published with the article. It must therefore be completed in full sentences and contain the exact wording you wish to be published.
a. State what role the funders took in the study. If the funders had no role in your study, please state: “The funders had no role in study design, data collection and analysis, decision to publish, or preparation of the manuscript.”

This work was co-funded by The Medical Research Council and Arts and Humanities Research Council (MC_PC_MR/R024510/1). TR, AS, JS and CH are funded through the ASSET research programme, supported by the UK's National Institute of Health Research (NIHR) (NIHR Global Health Research Unit on Health Systems Strengthening in Sub-Saharan Africa at King's College London (16/136/54)) using UK aid from the UK Government. CH also receives support from NIHR through grant NIHR200842 from AMARI as part of the DELTAS Africa Initiative [DEL- [15-01]. JS is an NIHR Senior Investigator and is supported by the National Institute for Health Research (NIHR) Applied Research Collaboration South London (NIHR ARC South London) at King’s College Hospital NHS Foundation Trust. The views expressed in this publication are those of the authors and not necessarily those of the NHS, the National Institute for Health Research or the Department of Health and Social Care, England. The funders had no role in study design, data collection and analysis, decision to publish, or preparation of the manuscript.

4. Please update the Funding Information to matched with Financial Disclosure Statement.

5.In the online submission form, you indicated that "The data generated and analysed during this study are not publicly available to protect individual anonymity but may be available from the corresponding author on reasonable request.". All PLOS journals now require all data underlying the findings described in their manuscript to be freely available to other researchers, either 1. In a public repository, 2. Within the manuscript itself, or 3. Uploaded as supplementary information.

Uploaded as Supplementary Information files 2 - 4.

6. Figure 3 includes an image of an identifiable person. Please provide written confirmation or release forms, signed by the subject(s) (or their guardian), giving permission to be photographed and to have their images published under a Creative Commons license. You may upload permission forms to your submission file inventory as item type 'Other'. Otherwise, we kindly request that you remove the photograph.

Figure 3 has been removed.
